# Evaluation of the long-term functional outcome and quality of life after rotationplasty in the management of primary malignant bone tumors about the knee in Children

**DOI:** 10.1051/sicotj/2026023

**Published:** 2026-05-12

**Authors:** Hazem Wafa, Walid Ebeid, Lennart Scheys, Lieven Moke

**Affiliations:** 1 Orthopaedic Surgery Department, Leuven University Hospital Leuven 3000 Belgium; 2 Institute for Orthopaedic Research and Training, Department of Development and Regeneration KU Leuven 3000d Belgium; 3 Orthopaedic Surgery Department, Cairo University Agouza Giza Egypt

**Keywords:** Rotationplasty, Limb salvage, Bone sarcoma, Quality of life

## Abstract

*Introduction*: Rotationplasty is a valid surgical technique in the management of bone sarcomas about the knee in children. This technique is often useful in patients with large extraosseous tumor extension and in the very young patients with anticipated significant limb length discrepancy. The aim of this study is to assess the long-term functional outcome and quality of life in our cohort of patients who have survived into adulthood. *Methods*: We have prospectively analyzed the functional outcome and quality of life in ten rotationplasty survivors. There were five male and five female patients with a mean age at the time of the index procedure of 11.6 ± 2.7 years. The functional outcome was evaluated using the Musculoskeletal Tumor Society Score (MSTS) and the Quality of Life (QoL) was assessed using the core quality of life questionnaire (QLQ-C30) of the EORTC. *Results*: Patients were followed up for a mean duration of 13.8 ± 4.4 years. The mean overall MSTS functional score was 82.7%. Nine patients reported either no or only minor functional restrictions. Only one patient used a crutch on walking for long distances, while other patients had unlimited or mild limitation of the distance that they could walk. The mean score of the global health status (QoL) was 85.8% with a mean score for social functioning of 90% and a mean score for role functioning of 88.3%. *Conclusions*: Rotationplasty affords the patients with an active lifestyle with no psychological or psychosocial disadvantages. The long-term assessment confirms that these patients maintain excellent functional results and quality of life through adulthood.

## Introduction

Rotationplasty is a segmental amputation with shortening of the lower extremity and rotation of the ankle 180° to function as a knee. This rotated foot is fitted to an exoprosthesis with a socket containing a plantar shelf to permit full weight bearing at the foot. The gastrocnemius muscle becomes the knee extensor while the anterior muscles of the leg act as knee flexors [1–3].

Rotationplasty is a valid technique in the management of primary malignant bone tumors of the lower extremity. The technique is often useful in patients with large extraosseous tumor extension precluding limb salvage surgery and in the very young patients with anticipated significant limb length discrepancy [4]. Its application as an alternative to above-knee amputation in the treatment of osteosarcoma of the distal femur was reported by Kotz and Salzer in 1974 [5]. de Bari *et al* [6] and Winkelmann [7] have described modifications of the rotationplasty for lesions of the proximal tibia. The prerequisites for this technique are a sciatic nerve free of tumor, and a disease-free ankle with a normal nerve and blood supply [2, 8, 9].

The parameters of motion, power, stability, and the adaptive phasic activity of the rotated muscles combine to give a characteristic gait pattern. Although their self-selected gait speed is reduced compared to that of healthy subjects, the visual impression of gait is not unduly altered. Acceptable locomotor function during gait, characterized by active control of the knee as well a smooth and coordinated gait pattern, has been reported [1, 3]. More detailed gait analyses furthermore reported functional flexion-extension patterns throughout the gait cycle, including initial flexion at heel strike, an apparent shock absorption pattern during weight acceptance, and flexion equal to or greater than the contralateral limb throughout the swing phase. Electromyographically, the function of the muscles of the involved limb was comparable to that of the uninvolved side. Overall, the gait characteristics were found to be comparable to those of patients who underwent a below-knee amputation or endoprosthetic reconstruction, and surpass the level of functionality associated with above-knee amputation. Young age at the time of surgery was found to be related to better gait and walking ability [1, 3].

Several studies reported lower energy cost with rotationplasty than above-knee amputation. Rotationplasty patients could walk for longer distances and they reported more active participation in sports and other physically demanding activities [1, 9, 10]. The functional results for these patients were found to be comparable with those for the patients who have an endoprosthesis. The presence of a biologic functional joint at the level of the knee contributes to the high level of function in these patients, as compared to patients with below-knee amputations. They also have the advantage of a natural weight-bearing foot with its superficial and proprioceptive sensibility [1, 4, 9, 10]. The aim of this study is to assess the long-term functional outcome and quality of life in our cohort of patients who have survived into adulthood.

## Material and methods

In this study we have prospectively analyzed ten rotationplasty survivors with primary malignant bone tumors around the knee joint. Ethical approval was obtained prior to data collection.

All patients included in this study were operated upon during the period from the 1st of October 2002 to the 1st of October 2004. During this period, we have performed 73 limb salvage operations for skeletally immature patients with primary malignant bone tumors around the knee, twelve of those underwent a rotationplasty procedure. Rotationplasty was our preferred method of reconstruction in those patients whose anticipated limb length discrepancy is > 5 cm by the end of skeletal growth. In those patients, lengthening of the limb could be performed at the time of the operation so that the level of the joints would be the same at the end of growth. We have also offered rotationplasty as an alternative to above-the knee amputation in those patients where limb salvage surgery could not be performed due to large soft tissue tumor extension with involving the main vessels.

In total 10 patients were enrolled in our study. There were five male and five female patients. The mean age of the patients at the time of the index procedure was 11.6 ± 2.7 years (median age, 11.5 years, range 7–16). The histological diagnoses included 9 osteogenic sarcomas, and one case of Ewing’s sarcoma. In this study the distal femur was involved in nine cases, whereas the proximal tibia was the site of involvement in one case. Exclusion criteria included patients who died with disease progression (two patients), and patients with major complications at the latest follow-up precluding functional evaluation and quality of life assessment.

The patients and their parents shared in the choice of the method of reconstruction. During the preoperative visit, the patients and their families were informed about the different possible reconstructive options in their case including the nature of the operation, the possible complications, the possibilities of requiring additional surgical interventions, the postoperative function, and the appearance of the limb. Patients considering the rotationplasty procedure were shown videotape examples and were offered a meeting with prospective patients to become aware of the appearance of the limb and the functional advantage of this procedure. Rotationplasty type AI was used for lesions in the distal femur while proximal tibial lesions needed to be treated by type AII according to the classification of Winkelmann [7].

### Technical tips

- The decision is made regarding the level of the femoral osteotomy so that at the end of growth, the rotated ankle joint would be at the same level as that of the contralateral knee. We lengthened the extremity by 4 cm in a 10-year-old child and 5 cm in an 8-year-old child to compensate for the difference in growth between the distal tibial physis of the operated extremity and the distal femoral physis of the contralateral extremity.
- The skin incision was made in the shape of a rhombus with its long axis on the anterior aspect of the lower limb. The proximal circumferential incision was made more proximal anteriorly, while the distal circumferential incision was made more distal anteriorly. The two circumferential incisions were connected by a posterior longitudinal incision.
- The superficial femoral vessels were identified proximally in the adductor canal, carefully dissected out and protected. They were traced distally into the popliteal fossa down to the level of the trifurcation while ligating the segmental branches to the gastrocnemius muscle and the posterior knee to avoid kinking and tension on the vessels when mobilized and rotated.
- The sciatic nerve and its two terminal branches: the tibial and common peroneal nerves were carefully dissected out and protected.
- If the femoral artery and vein were involved in a soft-tissue extension of the tumor, they were resected *en bloc* with it. They femoral vessels were identified proximal to the level of the femoral osteotomy and distally in the popliteal space. They were not ligated and divided until just before the specimen was removed in order to minimize the ischemia time of the distal leg.
- The distal extremity was externally rotated 180° and the distal femur and proximal tibia were brought together to confirm the proper length of the extremity according to the calculated lengthening required. Internal rotation was avoided to prevent stretching of the peroneal nerve.
- The sciatic nerve and femoral vessels were coiled medially between the muscles to avoid vascular kinking. If part of the vessels was resected, re-anastomosis was only performed after the osteosynthesis had been completed.
- The femur and tibia were joined together by means of compression-plate osteosynthesis.
- Soft tissue reconstruction was then performed by suturing the rectus femoris and vastus medialis muscles to the Achilles tendon, the vastus lateralis and vastus intermedius muscles to the tibialis posterior and toe flexors, while the hamstrings were sutured to the tibialis anterior and the toe extensors posteriorly. This was all done while the foot was held in plantigrade position.

Functional evaluation was performed using the Musculoskeletal Tumor Society Score (MSTS) described by Enneking *et al* [11]. This score is based on six categories – pain, level of activity and restriction, emotional acceptance, use of orthopedic supports, walking ability, and gait – with a maximum of five points for each category, and a sum-score out of thirty. The sum-score is transformed to a percentage scale.

Quality of life (QoL) was assessed using the standard version (version 3.0.) of the core quality-of-life questionnaire (QLQ-C30) of the European Organization for Research and Treatment of Cancer (EORTC) [12]. The questionnaires were completed by the patients during their follow-up appointments, then analyzed, and subscale scores were transformed to 0–100 scales using the scoring guidelines provided by the EORTC [13]. A high score for a functional scale represents a high level of functioning, a high score for the global health status represents a high QoL, while a high score for a symptom scale represents a high level of symptomatology.

### Methods of statistical analysis

Descriptive statistics were performed for all variables. Results are shown as mean (*M*) ± standard deviation (*s.d.*). All calculations were performed using Minitab® Statistical Software for Windows Release 13.1. (Minitab Inc., State College PA, USA).

## Results

Patients were followed up for a mean duration of 13.8 ± 4.4 years (median, 12.8 years; range, 9-23.2 years). All patients were fully weight-bearing on the affected extremity, and all were free from disease at the time of the latest follow-up. The mean range of motion of the ankle joint was 83° (range, 60°–90°), and the mean overall MSTS functional score was 82.7% (24.8 ± 3.54 points).

### Pain

Nine of the ten patients had either no or only slight pain and did not need any analgesic medication, with a mean score of 4.80 ± 0.36 points. The only patient who used non-narcotic analgesics was due to pain related to a poorly fitting exoprosthesis with skin ulceration of the foot.

### Functional activities

Six patients (60%) did not report any functional restriction and were able to participate in all activities they were involved in prior to their illness while another three patients reported only minor functional restrictions. Patients had a high score for functional activities (median, 5.0 points; mean and standard deviation, 4.50 ± 0.67 points).

### Emotional acceptance

Patients were asked whether they would recommend the same procedure to other patients and whether they would choose to have the same procedure under similar circumstances. Only one patient was hesitant about recommending the same procedure to others (a score of 3 points). The mean score for emotional acceptance was 4.50 ± 0.67 points.

### Supports

**Figure 1:**
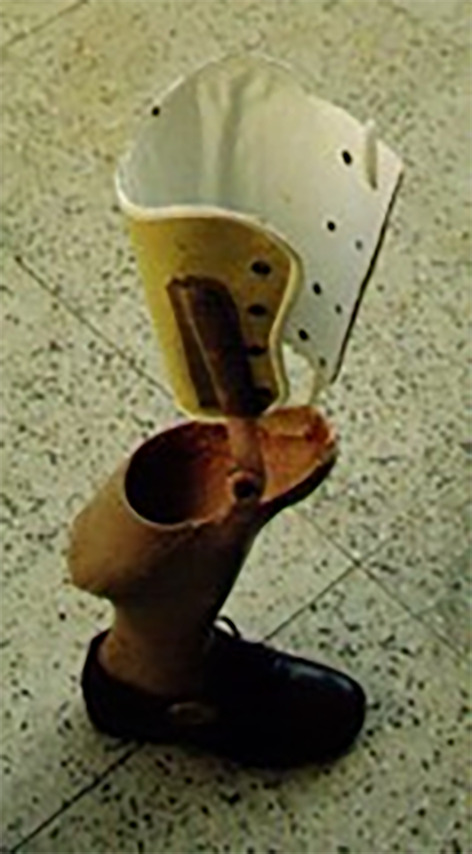
The rotationplasty exoprosthesis.

All patients wore the exoprosthesis (Figure 1) during their activities of daily living and only one patient used a crutch in addition to the exoprosthesis on walking for long distances.

**Figure 2:**
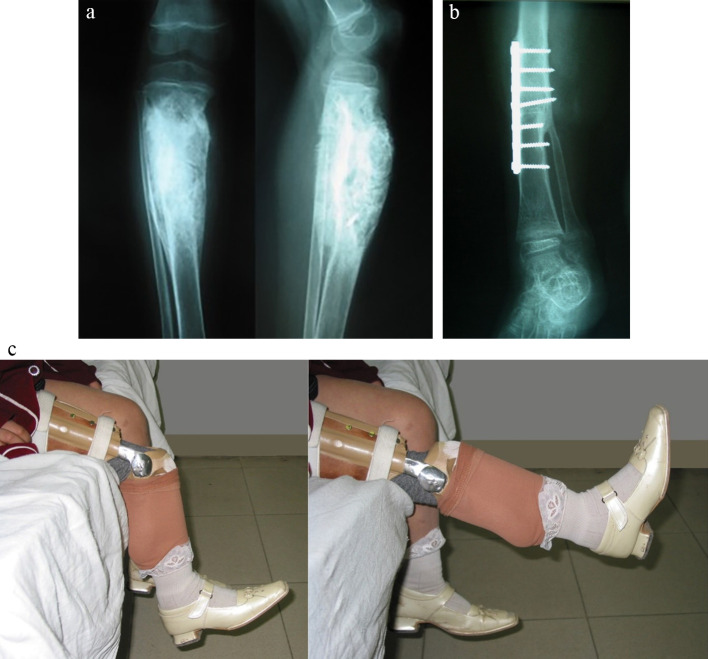
A seven-year-old girl with osteosarcoma of the proximal tibia who underwent rotationplasty type A II. Bony consolidation was seen at 3 months postoperatively. She maintained an excellent functional outcome and quality of life into adulthood with an MSTS functional score of 93.33% and a global quality of life score of 91.67%. 2-A Preoperative plain radiograph. 2-B Plain radiograph showing union of the tibia to the femoral stump. 2-C Photographs taken with the prosthesis fitted in place showing an active range of motion of the “knee” of 85°.

### Walking ability

At their latest follow-up, nine patients had unlimited or mild limitation of the distance that they could walk (a score of 4 or 5 points).

### Gait

Patients had a median score of 3.5 points (mean and standard deviation, 3.70 ± 1.00 points). Four patients had a score of 3, i.e., minor cosmetic alteration, while three patients had a score of 5, i.e., normal gait.

### Quality-of-life analysis

Our patients had a mean score of the global health status (QoL) of 85.83 ± 14.46 points. Physical functioning was assessed by questions about the ability to do strenuous activities like carrying heavy objects, taking a long walk or a short walk outside of the house, the need to stay in bed or a chair during the day, and the need for help with eating, dressing, washing themselves or using the toilet. The mean score for physical functioning was 84.33 ± 14.15 points (median 86.67 points).

Questions about limitations in doing work, daily activities, and pursuing hobbies and other leisure time activities were used to assess role functioning. The mean score for role functioning was 88.33 ± 21.15 points (median 100 points). Seven patients (70%) had no limitations in performing different activities (a role functioning score of 100 points).

Social functioning was assessed by asking the patients whether their physical condition or medical treatment had interfered with their family life and social activities during the week prior to completion of the questionnaire. The mean score was 90 ± 13.33 points.

The emotional functioning scale consists of four items regarding depression, tension, irritability, and worry. Our patients had a mean score of 90.83 ± 11.46 points.

All of our patients reported no or very minor issues with their abilities to concentrate or remember things with a mean score for cognitive functioning of 96.67 ± 6.67 points.

Patients were asked whether they had any pain and whether this pain had interfered with their daily activities over the last week prior to the completion of the QoL questionnaire. The mean score for pain was 11.67 ± 16.75 points while the mean score for fatigue was 12.22 ± 16.82 points. No restriction or only mild restriction in daily activities secondary to pain (a score of 0 to 33.3 points) was reported by nine (90%) of the patients.

## Discussion

Rotationplasty is a safe alternative to amputation in the management of malignant tumors about the knee joint. This procedure is also useful in the young children with anticipated significant leg length discrepancy after limb salvage surgery. There has been a major shift towards the use of expandable megaprostheses in those patients. However, multiple revision surgeries seem inevitable, and the lifelong risk of developing periprosthetic infection is a serious concern in these patients. Multiple studies have reported excellent functional results and quality of life in patients who have undergone rotationplasty. Our study was focused on the long-term outcome of rotationplasty in patients who underwent this procedure in childhood (range, 7–16 years) with the aim to assess whether they have maintained their excellent functional outcome and quality of life through adolescence into young adulthood. Our cohort of rotationplasty patients had a mean MSTS functional score of 82% at a mean follow-up of 13.8 years. Our functional results match those reported by Benedetti *et al* [14] with a mean MSTS functional score of 83.3% at a mean follow-up of 15 years. None of our patients demonstrated evidence of functional decline on the long term and the majority (90%) reported either no or only mild limitation of the distance they could walk. Hillman *et al* [15] reported no significant difference in the functional outcome between the patients who had an endoprosthetic replacement and those who had a rotationplasty. However, they reported a restriction in activities related to work in 18% of the patients who had an endoprosthesis as compared to only 3% of the patients who had a rotationplasty. Another study by Hopyan and colleagues [16] reported a minimal evidence of greater physical activity among patients who have undergone rotationplasty compared to both the amputation and limb-salvage groups of patients.

The perceived quality of life by the patients often differs from the reported functional outcome by the treating physician which is usually based on clinical and radiological assessments in the outpatient clinic while the perceived quality of life is also influenced by other factors including self-image, emotional acceptance, and social relations. While physical functioning assesses the patient’s ability to do different physical activities like carrying heavy objects, walking, and performing basic activities especially those related to personal care, eating and dressing, role functioning assesses the patient’s capacity for doing work, pursuing hobbies, and other leisure time activities. Multiple studies have shown that rotationplasty has afforded patients active lifestyles and less use of assistive devices, and recipients have compared well with those who have undergone endoprosthetic reconstructions and who have other chronic illnesses [8, 9]. It has also been reported that rotationplasty patients could participate in active sports, which is rare in patients with endoprostheses [5, 8, 9, 14, 17, 18]. Our patients reported minor restrictions with daily activities and the majority (70%) reported no restrictions in performing different activities. They were not protective about their limbs and one patient even broke his exoprosthesis while playing soccer. Our findings match those of Gradl *et al* [19] who reported only minor restrictions in participation in sports activities in 83.3% of their 12 rotationplasty survivors at a mean followup of 14 ± 9 years.

The cosmetic results of the rotationplasty and its possible adverse sequelae had always been a major concern in the literature. Our patients were satisfied with the appearance of the limb. We believe that they did benefited a lot from meeting other patients who had had this procedure before. None of our patients had expressed regret at having had the operation, and none of them had reported any psychological problems. This is in line with the literature with multiple studies reporting no negative effects of the rotationplasty procedure on the psychological and psychosocial functioning of patients [5, 6, 8, 9, 18, 20]. Kotz *et al* [5] have reported that none of their rotationplasty children have complained of any problems in school due to the unusual appearance of the lower limb and foot, or have found it necessary to hide the handicap from friends. Hanlon and Krajbich [18] reported that all patients stated that their friends and family had adjusted to the appearance of the limb, and no patient thought that the appearance of the limb affected their ability to pursue recreational, educational, or career goals. Gradl *et al* [19] reported that all but one of their patients (11/12) would choose rotationplasty again as a treatment option over amputation or endoprosthetic reconstruction.

Our rotationplasty survivors did not report any negative influence of their physical condition, body image, and treatment on their family lives and social relationships. This could possibly explain their excellent role functioning scores and the absence of any negative effect of the rotationplasty procedure on their psychological functioning. We have not observed any significant differences between our male and our female patients with regard to participation in sports activities, and our patients maintained their high social, emotional and role functioning scores through adolescence to adulthood. These findings are different from those reported by Forni *et al* [21] with the greater well-being in the Mental Component Summary of the generic SF36 health questionnaire in their patients aged over 24 being attributed to relational and emotional difficulties in adolescence, which were partially overcome in adulthood. They have also identified a trend for wider participation of their male patients in sports activities. On the other hand, Jacobs *et al* [20] stated that their rotationplasty patients adapted exceedingly well to the procedure and were anxious to be active without any intersocial or psychological problems. Moreover, another study even reported a positive influence of the rotationplasty procedure on their patients [8]. This included increased self-confidence with patients enjoying life more intensely, being better able to put life events into perspective, and having better relationships.

## Conclusions

Rotationplasty is a useful technique in the management of malignant bone tumors about the knee. This technique affords patients with an active lifestyle with no psychological or psychosocial disadvantages. The long-term assessment confirms that these patients maintain excellent functional results and quality of life through adulthood.

## Data Availability

Anonymized data can be provided by the corresponding author upon request.
